# The Effect of Conjugated Bilirubin on the Measurement of Unbound Bilirubin

**DOI:** 10.1111/apa.70357

**Published:** 2025-10-27

**Authors:** Alan Kleinfeld, Andrew Huber, William Oh, Thomas Hegyi

**Affiliations:** ^1^ Fluoresprobe Sciences, Inc. San Diego California USA; ^2^ Department of Pediatrics Warren Alpert Medical School of Brown University Providence Rhode Island USA; ^3^ Division of Neonatology, Department of Pediatrics Robert Wood Johnson Medical School, Rutgers, The State University of New Jersey New Brunswick New Jersey USA

**Keywords:** direct bilirubin, UBCheck, unbound bilirubin

## Abstract

**Aim:**

Unbound bilirubin (Bf) is a stronger predictor of neurotoxicity in newborns than that of total serum bilirubin (TSB). Standard Bf assays rely on peroxidase methods (e.g., Arrows analyser), whereas UBCheck employs a near‐infrared fluorescence sensor. Because conjugated bilirubin (CBR) levels are often elevated in infants, they may alter Bf measurement accuracy, particularly with peroxidase‐based methods. This study compared CBR effects on Bf measurement results from the UBCheck and Arrows assays and evaluated whether they reflected albumin displacement, assay interference, or both.

**Methods:**

Bilirubin–albumin complexes were spiked with CBR (0.1–5 mg/dL). Bf was measured with both assays using a three‐stage paired‐sample protocol to assess displacement and interference.

**Results:**

At CBR ≤ 0.2 mg/dL, both assays yielded comparable Bf values. At 0.5–3 mg/dL, Arrows showed 12%–120% Bf increases, while UBCheck remained stable. At 4 mg/dL, UBCheck rose 20%, and Arrows increased by 220%. High CBR acted as both interferent and displacer.

**Conclusions:**

In the presence of neonatal CBR concentrations, UBCheck provides reliable Bf measurements, whereas Arrows substantially overestimates Bf, limiting its clinical utility.

**Abbreviations**
Bf: unbound bilirubin
BT: total bilirubin
CBR: conjugated bilirubin
diTBR: ditaurobilirubin

**Summary**

- The effect of conjugated bilirubin (CBR) levels on unbound bilirubin (Bf) measurements using UBCheck and Arrows assays was compared.
- At CBR levels up to 0.2 mg/dL, Bf measurements by both methods were not affected. However, at higher levels, the Arrows method was subject to an overestimation of Bf levels, whereas the UBCheck was not.

## 1 Introduction

Unbound bilirubin (Bf) is the neurotoxic fraction of bilirubin capable of crossing the blood–brain barrier and damaging neurons. In vulnerable neonatal populations, particularly preterm infants, the risk of bilirubin‐induced neurologic dysfunction (BIND) and kernicterus correlates more strongly with Bf levels than with those of total serum bilirubin (TSB) [1, 2, 3]. While the majority of bilirubin is tightly bound to human serum albumin (HSA), a small, dynamic fraction remains unbound. This Bf pool can increase substantially under conditions that reduce binding affinity to albumin, such as drug displacers, elevated free fatty acid levels, acidosis, or hypoalbuminaemia in newborns [4, 5, 6].

Historically, the peroxidase method has been the mainstay for measuring bilirubin binding and displacement. Although it has provided critical insights, the technique requires significant dilution of plasma samples, which shifts the bilirubin–albumin equilibrium toward tighter binding, thereby underestimating the degree of displacement [7, 8, 9]. To address this limitation, the innovative UBCheck system has been developed, which utilises a new near‐infrared fluorescent probe that allows quick and direct measurements of Bf in undiluted whole blood [10]. Early proof‐of‐concept studies demonstrated that the device could rapidly and reliably quantify Bf, overcoming the dilutional artefacts inherent in the peroxidase method [11]. Subsequent work validated the device against clinical samples, demonstrating superior precision and reproducibility, and highlighted its potential for guiding the management of neonatal hyperbilirubinaemia, as well as identifying infants at risk of developing bilirubin neurotoxicity [12, 13, 14].

Although the majority of hyperbilirubinaemic neonates present with elevated unconjugated bilirubin, a subset exhibits increased conjugated bilirubin (CBR) levels, including those with congenital abnormalities, infections, metabolic disorders, iatrogenic exposures and idiopathic conditions [15]. For these infants, Bf measurements using the peroxidase method are particularly problematic, as the method can produce artifactually elevated Bf values [16]. The present study was therefore designed to determine whether CBR can also interfere with UBCheck measurements, and if so, whether the effect is due to true bilirubin displacement from albumin, direct assay interference, or both.

## 2 Methods

To determine whether CBR affects Bf levels measured by the Arrows (Arrows Co., Osaka, Japan) or UBCheck (Fluoresprobe Sciences, San Diego, CA) methods, we employed a three‐stage protocol designed to distinguish bilirubin displacement from assay interference. Paired serum samples were prepared from pooled neonatal serum; one aliquot was spiked with CBR at defined concentrations, and the paired control with solvent ditaurobilirubin (diTBR), eliminating variability in baseline bilirubin binding. A ≥ 10% change in Bf relative to the control was prespecified as significant.

In Stage 1, pooled neonatal serum (albumin 3.5–4.5 g/dL) was supplemented with bilirubin to achieve clinically relevant TSB (10–20 mg/dL), then exposed to CBR (0.5–25 μM). Samples were assayed in duplicate without dilution to preserve physiologic binding. Both the Arrows (peroxidase–bilirubin oxidation) and UBCheck (fluorescent displacement) methods were performed according to the manufacturer's instructions.

In Stage 2, bilirubin‐free neonatal serum (charcoal‐stripped) was used to isolate direct assay effects, allowing for the detection of nonspecific UBCheck fluorescence or Arrow peroxidase signal shifts attributable to interference.

In Stage 3, compounds active in Stages 1 and 2 were further characterised. Agents that increased Bf only in Stage 1 were classified as bilirubin displacers, whereas those that also altered assay behaviour in bilirubin‐free serum were designated as interferents, with or without combined displacement effects.

All samples were equilibrated at 37°C for 30 min before analysis, and results were averaged from triplicates with coefficients of variation < 5%.

### 2.1 Displacement Assay

Bilirubin–albumin complexes were added to phosphate‐buffered saline (pH 7.4, albumin 40 g/L) to yield Bf concentrations of 10–12 nM in the first run and 30–32 nM in the second, without added CBR. The samples were then exposed to diTBR at concentrations ranging from 0.05 to 20 mg/dL. Bf was measured in each sample using the Arrows and UBCheck devices. The UBCheck sensor contains a Bf‐specific fluorescent dye with a strong, reversible binding affinity. The fluorescence at 700 nm is quenched when bilirubin binds, while the signal at 819 nm remains unchanged. Bf is calculated from the ratio of fluorescence intensities at 700 nm to 819 nm [11].

### 2.2 Assay Interference Test

To evaluate potential CBR interference with the UBCheck sensor, each spiked sample was added to a solution containing a known concentration of Bf in an aqueous buffer without albumin. A decrease in fluorescence indicates that the drug might displace bilirubin or interact with the dye, suggesting interference with the assay. The difference greater than 2.8 nM between UBCheck Assay median values for test and control samples was considered significant. The 2.8 nM threshold was established based on the lower reportable range of the UBCheck Assay.

### 2.3 Dose–Response Studies

Samples for the dose–response titration experiments were mixtures of two 20 nM UB serum stocks: A 0% interferent stock and a 100% interferent stock. The 100% interferent stock concentration was set to be the lowest concentration known to cause significant interference with UBCheck measurements. Mixtures of 50% were made. Seven replicate measurements were made at each concentration.

## 3 Results

CBR consistently produced increases in Bf exceeding the 10% threshold, with differences greater than 2.8 nM in later testing, confirming its classification as an interferent across all conditions, including serum with a baseline Bf of 7 nM.

### 3.1 Bilirubin Displacement

**FIGURE 1:**
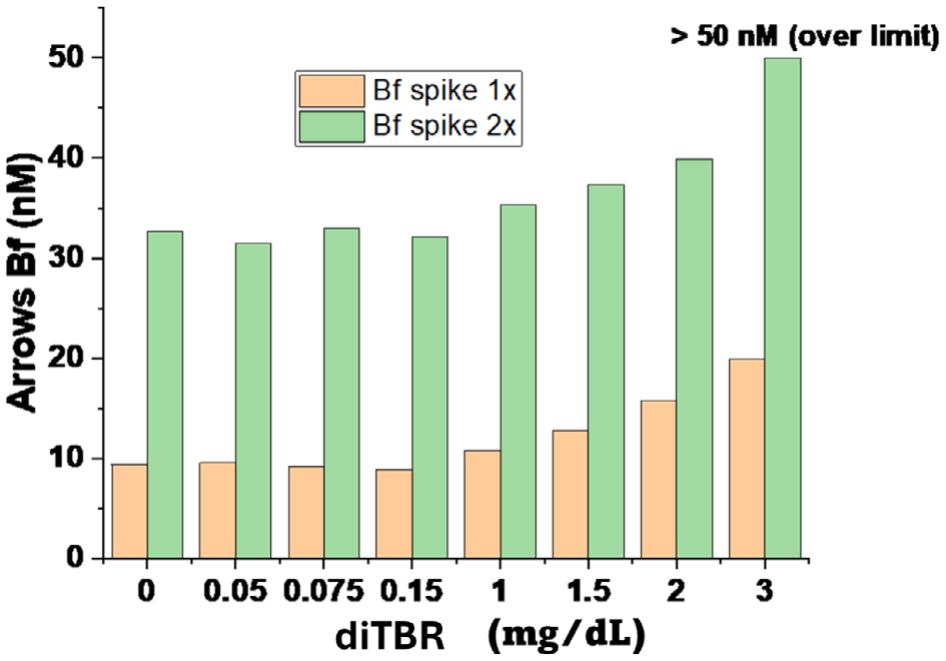
Arrows measurements of Bf in serum spiked with increasing CBR concentrations. Serum was spiked (1×) to yield 10 nM and 2×, yielding 32 nM Bf. Both samples were titrated with increasing concentrations of CBR (diTBR). Arrows Bf measurement began to rise at CBR ≥ 1 mg/dL.

**FIGURE 2:**
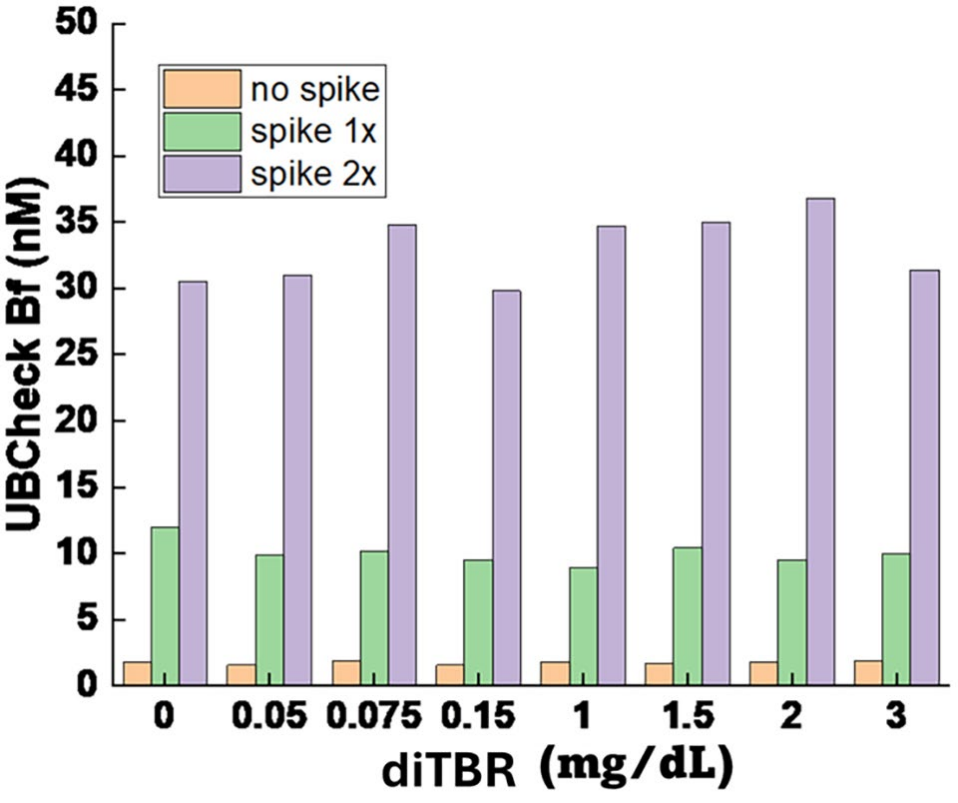
UBCheck measurements of Bf serum spiked with increasing CBR concentrations. Serum was spiked (1×) to yield 12 nM and 2×, yielding 30 nM Bf. Increasing concentrations of CBR (diTBR) were added to both samples. UBCheck Bf measurements were not affected by CBR levels up to 3 mg/dL.

Both Bf concentrations measured with both the Arrows and UBCheck methods remained unchanged at CBR concentrations ≤ 0.2 mg/dL, with baseline values of ~10 nM. However, clear differences emerged at higher concentrations. Between 0.5 and 3 mg/dL, Arrows measurements showed a progressive increase, with Bf rising by 12%–120% relative to baseline (Figure 1). By contrast, UBCheck values at the same concentrations showed no increase in Bf at either 12 or 30 nM baseline levels (Figure 2).

**FIGURE 3:**
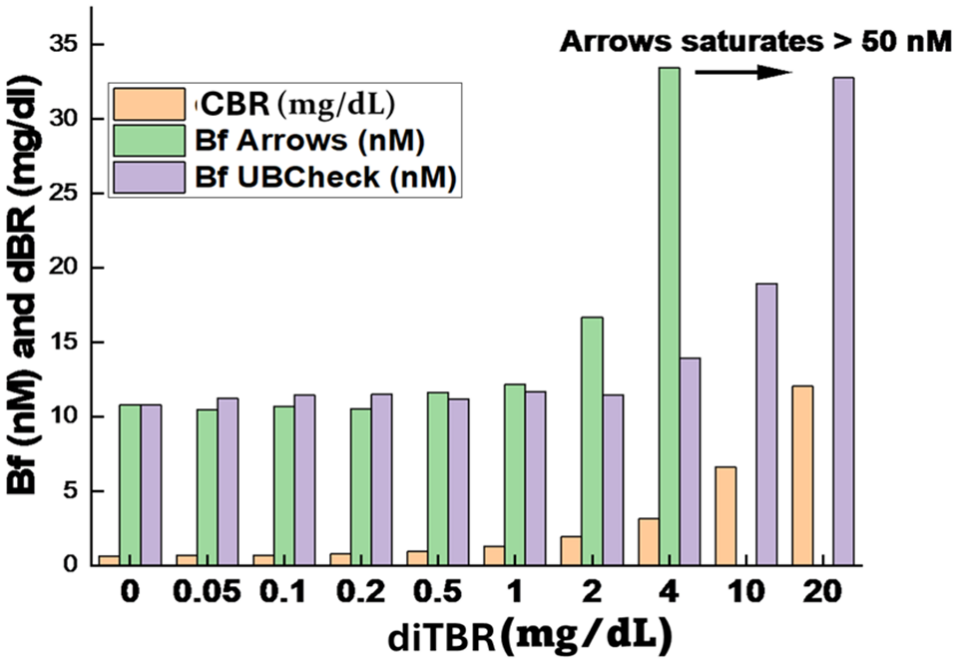
Comparison of Arrows and UBCheck measurements of Bf in serum with increasing CBR concentrations. Serum was spiked to yield 10 nM Bf, and then increasing concentrations of CBR (diTBR) were added up to 20 mg/dL. The Arrows measurement began to overestimate Bf levels at a CBR level of 1 mg/dL, rising to 4 mg/dL, and then saturating at a Bf level above 50 nM. In contrast, the UBCheck Bf began to increase at a CBR concentration of 4 mg/dL.

At high CBR concentrations (up to 20 mg/dL), Bf levels measured by the two assays diverged further (Figure 3). Arrows Bf measurements began to increase at 1 mg/dL of CBR, reaching 35 nM by 4 mg/dL, and plateaued above 50 nM of Bf. Bf levels measured by UBCheck, in contrast, showed no increase until ~4 mg/dL of CBR, after which Bf levels rose steeply, exceeding 200 nM at higher concentrations of CBR.

### 3.2 Assay Interference

CBR acted as both an interferent and a displacer of bilirubin from albumin when using the UBCheck assay. In Stage 1 screening at low baseline Bf levels, gentamicin served as a comparator and showed a modest reduction (−3.6%), whereas CBR caused an increase of nearly 5000%. At higher baseline Bf levels, gentamicin again showed a negligible effect (−0.8%), while CBR increased Bf levels by over 5600%. In Stage 2 confirmatory testing, CBR produced a mean Bf of 3.01 nM, representing a 2.99 nM difference from control—exceeding the advancement threshold. In Stage 3 testing, the addition of CBR increased mean Bf to 11.97 nM, which had an 8.98 nM displacement relative to control.

### 3.3 Dose–Response Results

**FIGURE 4:**
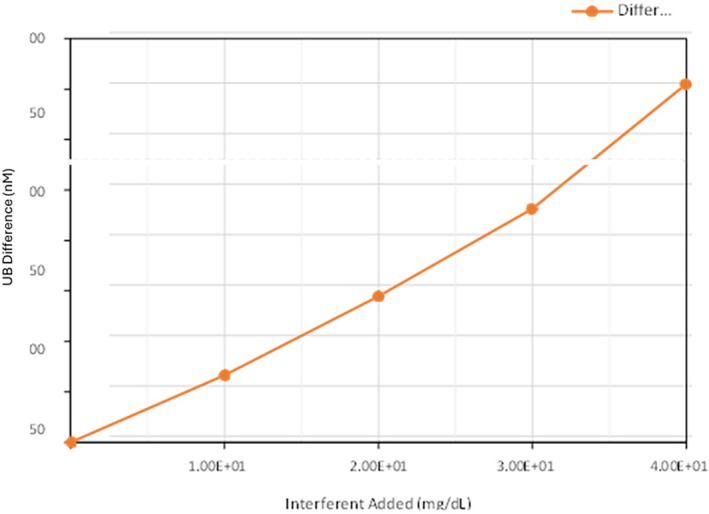
CBR dose response. The cutoff for significant interference in blanked serum is a 2.8 nM change in UBCheck Assay UB value relative to the 0% interferent UB value. A 2.8 nM increase in UB (nM) is predicted to occur between 22.5 (75%) and 30.0 mg/dL (100%) CBR. The slope of the line between these two points is 0.1239, and a 2.8 nM increase in UB value is estimated to occur at 33.9 mg/dL CBR.

Dose–response experiments demonstrated that CBR interference occurred at higher concentrations, with significant effects observed at 33.9 mg/dL (Figure 4). The magnitude of displacement and interference confirmed that CBR exerts a strong dose‐dependent effect on Bf measurements, particularly when using the Arrows assay.

## 4 Discussion

The UBCheck device is a substantial advancement in Bf measurements, demonstrating accuracy and reliability, particularly at CBR concentrations below 4 mg/dL. In contrast, CBR levels affected the Arrows method, which overestimated Bf levels at CBR concentrations typically found in newborns. Accurate measurements of Bf are crucial because it is the only bilirubin form that crosses the blood–brain barrier, enters the brain and causes neurotoxic effects [1, 17]. A limitation of this study is that it was performed in vitro, and further testing in infants is needed to verify these results.

Bilirubin, a hydrophobic tetrapyrrole, is generated from the breakdown of haem. In plasma, most of the bilirubin is noncovalently and reversibly bound to serum albumin, which serves as the most common plasma protein and the primary carrier of bilirubin. This binding occurs at a high‐affinity site on albumin, known as Sudlow site I, characterised by a dissociation constant in the nanomolar range [18, 19]. The reversible nature of this interaction means that other substances, such as drugs, excipients, or metabolites that bind to the same or overlapping sites on albumin, can competitively displace bilirubin, thereby altering the Bf concentration [20, 21].

The effect of a compound on Bf levels can arise through two distinct mechanisms. First, a compound may interact directly with the assay method. In the case of the UBCheck assay, such an interaction can alter bilirubin binding within the device or interfere with its signalling chemistry. This constitutes analytical interference, resulting in erroneous Bf levels that do not accurately reflect the physiological state. Second, a compound may interact with HSA, competitively displacing bilirubin from its binding sites. This interaction leads to a true physiological increase in circulating Bf levels. In this scenario, elevated Bf level values reported by the UBCheck accurately reflect the biological state and may indicate a potentially hazardous risk for developing bilirubin neurotoxicity.

This distinction is clinically critical because increases in Bf caused by displacement, hypoalbuminaemia, or HSA mutations that reduce bilirubin affinity are often undetectable by TSB measurement alone. Thus, accurate determination of developing Bf levels is essential for identifying infants at greatest risk of bilirubin encephalopathy.

We found that CBR produced significant displacement of bilirubin from HSA, leading to marked increases in measured Bf, consistent with its capacity to compete for albumin binding. By contrast, gentamicin—an antibiotic with high polarity and negligible albumin binding—produced no significant effect on Bf, in agreement with its established pharmacokinetics and prior reports [19].

Iwatani and coworkers recently described a modification of the glucose oxidase–peroxidase (GOD–POD) assay that incorporates the bilirubin‐inducible fluorescent protein UnaG, allowing for the rapid kinetic measurement of Bf levels while eliminating the positive interference from direct (conjugated) bilirubin that limits conventional peroxidase methods [22]. In contrast, the UBCheck system is a point‐of‐care cartridge and reader platform that measures equilibrium Bf directly using a fluorescent binding‐protein sensor, thereby avoiding peroxidase‐related artefacts and requiring only microliter sample volumes with rapid turnaround. Both approaches represent advances over traditional peroxidase assays: the GOD–POD–UnaG method offers a laboratory‐based solution to the problem of CBR interference. However, UBCheck offers a direct approach for measuring Bf, as it quantifies equilibrium free bilirubin using a fluorescent binding‐protein sensor, thereby avoiding the indirect kinetic estimation and susceptibility to CBR interference inherent to peroxidase‐based methods.

A survey of diagnostic reagents likewise reported variable reactivity of in vitro bilirubin assays to CBR and photoisomers, emphasising that CBR can produce artifactually elevated Bf readings unless the assay discriminates between bilirubin species [23].

Two central findings emerge from this investigation. First, the Arrows peroxidase method is substantially affected by CBR, yielding artefactual increases in Bf at clinically relevant concentrations, whereas the UBCheck assay remained unaffected under the same conditions, except at supraphysiologic CBR concentrations that are unlikely to occur in newborns at risk for hyperbilirubinaemia [24]. Second, although UBCheck itself demonstrated sensitivity to very high CBR concentrations, the observed increases reflected a combination of bilirubin displacement from albumin and assay interference. In summary, UBCheck provides a more accurate and clinically useful measurement of Bf across the relevant physiological and pharmacological ranges, and thus an ideal method for identifying infants at risk of developing bilirubin neurotoxicity [25].

## Author Contributions

Drs. Hegyi, Huber, Oh and Kleinfeld contributed to all essential aspects of this study, including concept and design, data analysis and interpretation, drafting and revising the manuscript. All authors participated in the manuscript review, revision and approval of the version to be published.

## Conflicts of Interest

Dr. Kleinfeld is the CEO of Fluoresprobe Inc.

## Data Availability

Research data are not shared.
